# Clusters of cultures: diversity in meaning of family value and gender role items across Europe

**DOI:** 10.1007/s11135-016-0422-2

**Published:** 2016-10-11

**Authors:** Eva van Vlimmeren, Guy B. D. Moors, John P. T. M. Gelissen

**Affiliations:** 1 Department of Methodology and Statistics, Tilburg University, Tilburg, The Netherlands; 2 Department of Methodology and Statistics, School of Social and Behavioral Sciences, Tilburg University, PO Box 90153, 5000 LE Tilburg, The Netherlands

**Keywords:** Cross-cultural comparative research, Measurement invariance, Acquiescence, Cultural diversity, Gender roles, Family values

## Abstract

Survey data are often used to map cultural diversity by aggregating scores of attitude and value items across countries. However, this procedure only makes sense if the same concept is measured in all countries. In this study we argue that when (co)variances among sets of items are similar across countries, these countries share a common way of assigning meaning to the items. Clusters of cultures can then be observed by doing a cluster analysis on the (co)variance matrices of sets of related items. This study focuses on family values and gender role attitudes. We find four clusters of cultures that assign a distinct meaning to these items, especially in the case of gender roles. Some of these differences reflect response style behavior in the form of acquiescence. Adjusting for this style effect impacts on country comparisons hence demonstrating the usefulness of investigating the patterns of meaning given to sets of items prior to aggregating scores into cultural characteristics.

## Introduction

 To develop measurements of national cultures, scholars often use cross-national surveys and aggregate individual-level responses to Likert-type items from these surveys to the national level. For example, Inglehart (1997) positions countries on a survival versus self-expression dimension and a traditional versus rational-secular dimension by aggregating factor scores derived from individual-level measurements of personal values and attitudes. Other experts on human values systems, such as Schwartz (1992) or Hofstede (2001) also investigate cultural differences using aggregate scores derived from individual-level variables. However, the approach of using aggregated scores is prone to two complications that may confound findings in cross-cultural research: firstly, cultural diversity in the interpretation of the content of items and secondly, cultural differences in how people respond to survey questions regardless of the content of the questions.

The first complication is researched by checking measurement invariance (Kankaras and Moors 2010; Steenkamp and Baumgartner 1998; Vandenberg and Lance 2000). Establishing measurement invariance implies that all groups being compared assign the same meaning to all items that compose a measurement scale. However, tests of whether measurement invariance holds across groups usually lead to the conclusion that one cannot validly compare groups, and thus suggests that means scores across countries are not fully comparable. Recently this perspective has been challenged by Welzel and Inglehart (2016) who argued that even in cases that country level aggregate scores entirely lack convergence at the individual level, i.e. measurement in-equivalence, these aggregate scores still are meaningful in their own right when they converge with external criteria. In their study Welzel and Inglehart (2016) validate country-level scores on emancipative values with indices of effective democracy and cognitive mobilization. In this paper we reason differently. In our view finding out whether respondents from different countries assign the same meaning to survey items is a cultural finding in its own right and not merely a methodological artifact. Furthermore, we will show that neglecting this diversity in aggregating individual responses to the national level might be dangerous.

The second complication in cross-cultural research is the cultural difference in response styles. Respondents in certain countries can be influenced by certain cultural norms when answering survey questions, for example, by having a tendency to answer toward the agree-side of the scale, regardless of item content. Previous research (Billiet and McClendon 2000; Welkenhuysen-Gybels et al. 2003) has demonstrated that this tendency, also known as acquiescence, systematically distorts measurement. Usually this is regarded as a form of bias, but in this research we think of this phenomenon as another source of cultural diversity in responding to survey items. The mapping of such cultural variations in response style is of equal importance as the mapping of content variation.

In this paper we investigate whether, and to what extent, cultural variations exist in the meaning assigned to items linked to family values and gender role attitudes across Europe. In a European Union context in which egalitarian policies are developed, an increased understanding on what causes cultural variations in these items is of utmost importance. The novelty of our study involves following a stepwise approach aimed ad discovering the principal cultural differences. First, we use cluster analysis to group countries in such a way that the largest differences in (co)variances among both sets of items are identified in clusters of cultures. These clusters are internally more measurement invariant than the pooled group of European countries, thus increasing within cluster comparability. Then we estimate separate measurement models for all clusters. Each measurement model is then adjusted for acquiescent response behavior and compared to models that do not adjust for this type of response style. The final purpose then is to demonstrate how within ‘clusters of cultures’ differences between countries shift, depending on whether the measurement model has been defined on the pooled versus cluster specific measurement models. Whether accounting for acquiescence contributes to our understanding of differences is demonstrated as well. This study will show that particular family values and gender role items truly have different and sometimes opposite meanings in different countries, even to the extent that what is regarded as egalitarian in one culture might have an opposite meaning in another culture.

## Clusters of cultures

To identify national cultures, scholars can use survey data on values and attitudes and aggregate Likert-type scale scores to the national and/or regional level. To do this it is necessary that respondents assign a similar meaning to the questions that are used to measure these attitudes or values (Hui and Triandis 1985; Johnson 1998; Kankaras and Moors 2010). This is commonly known as measurement equivalence (Hui and Triandis 1985; Van de Vijver and Leung 1997) or measurement invariance (Welkenhuysen-Gybels et al. 2003). The assessment of measurement invariance usually follows a standard procedure, which involves the formal comparison of the (co-)variance matrices of all items across all countries in a series of measurement models defining different levels of comparability (Steenkamp and Baumgartner 1998; Vandenberg and Lance 2000). The initial step is an omnibus test of overall measurement invariance. It examines whether the observed covariance matrices are invariant (or similar) across all countries. In practice, the null hypothesis stating invariant observed covariances, indicating incomparability between countries, is usually rejected making the test virtually trivial. The next step is to check for configural invariance that evaluates whether the pattern of factor loadings is similar in every country. Configural invariance is required to test for metric invariance, that verifies whether factor loadings are similar or not, and for scalar invariance, that tests whether both the factor loadings and the regression intercepts of the observed items related to the latent variables are invariant across countries.

In comparative cross-cultural research, differences in these factor loadings mean that respondents from a certain country interpret the meaning of survey questions differently from respondents in another country. If measurements are not at least metric invariant across countries, then comparing these country means would be like comparing apples to oranges. Hence, the importance of assessing measurement invariance is evident as a universe of cultures is rarely established and procedures to identify countries that have comparable measurements do not always produce unequivocal results (Welkenhuysen-Gybels et al. 2003; Welkenhuysen-Gybels and Van de Vijver 2001).

In this study we deviate from the stepwise procedure by, in the first step of the analysis, clustering countries according to their similarities in their variances and covariances between items, resulting in subsets of countries in which measurement invariance is more likely to occur. The need to identify subsets of equivalent groups has been recognized by others (Welkenhuysen-Gybels et al. 2003; Welkenhuysen-Gybels and Van de Vijver 2001). Their approach involved a cluster analysis either on a similarity matrix for the factor loadings or on the factor loadings themselves, which is at the final step of the analyses. We also run a cluster analysis, but then on the associations between the items of the selected scales, which is prior to deciding on a particular measurement model. Consequently, we postpone the issue of defining a measurement model that is required to use in the standard procedure for studying measurement invariance. The key idea is that when countries share similar associations between items, they share measurement invariance regardless of how that measurement model could be defined. Following this logic, we cluster European countries into a smaller number of clusters of countries that are internally more homogeneous than the European sample as a whole. Comparing the differences in the measurement models between these clusters of countries then reveals the main source of heterogeneity in meaning assigned to the items.

Admittedly, this approach is exploratory, disregarding any theoretical reasoning regarding the underlying structure that causes the relationships among the items. However, this is exactly what we aim at: first we cluster countries with respect to their similarity in associations between items into sets of countries; then, in the next step of the analysis, we find the logic of the underlying measurement model within each set of countries defined. While we acknowledge that our procedure results in clusters of countries for which measurement invariance *between* clusters is not established, we emphasize that our interpretation of the results allows us to identify clusters of cultures that adopt different perspectives on the meaning of items. Hence, lack of measurement invariance is more than merely a methodological artifact: it also signals cultural variation in meaning giving systems. Thus, the goal of this paper is *not* to establish measurement invariance, but rather to elevate the concept of difference in meaning assigned to survey questions as an expression of cultural diversity. Checking whether and to what extent acquiescence affects the measurement model is part of identifying the underlying logic of the measurement model. Previous research (Welkenhuysen-Gybels et al. 2003) has demonstrated that including such a response style factor often increases the validity of measurements in assessments of measurement invariance. Before elaborating on modeling acquiescence we need to turn attention to the aforementioned recent polemic initiated by Welzel and Inglehart (2016) regarding the question whether establishing measurement invariance is truly necessary to aggregate individual level responses to the country level.

### Misconceptions of measurement invariance?

The three classical approaches in researching cultural differences mentioned in the introduction, i.e. Hofstede, Inglehart and Schwartz, share the same assumption that value orientations that characterize cultures can be inferred from averaging scores of individual in ‘matched samples’ from each society. Such samples might be either representative samples or selective homogenous samples. This assumption has been challenged by scholars (e.g. Davidov et al. 2012) in structural equation modeling who argue that valid comparisons are only justified when (scalar) measurement invariance is established. Among the three classics Schwartz is most likely the one who assigns the most importance to this issue by recognizing that “values whose meanings differ across cultures should not be used in cross-cultural comparison” (Schwartz 2006, p. 144). Hofstede’s perspective (2001) is different. He claims to present comparable results since his sampling design involves the selection of a highly homogenous group within countries, namely IBM workers. All these workers have experienced in-the-job training in an international company with a clear organizational culture. These workers were recruited on the same criteria thus increasing the likelihood that they are from similar educational background in all societies. As a consequence Hofstede argues that if any difference is observed between workers from different cultures this can only reflect true cultural differences. Inglehart (1997), on the other hand, makes use of large within country samples that aim at capturing the heterogeneity within and between societies. It is within this context that the ‘misconceptions of measurement equivalence’ have been articulated. In a nutshell Welzel and Inglehart (2016) argue that—paraphrasing their own words—convergence patterns at the aggregate (country) level exist in their own right even when constructs entirely lack convergence at the individual level. Convergence at the aggregate level refers to criterion-related external validation of country-level indices. Convergence at the individual level is synonym of measurement invariance. Hence, they do not feel a need for establishing measurement invariance. They propagate a ‘combinatory logic’ as opposed to the ‘dimensional logic’ of the latent variable modeling approach. This combinatory logic involves the theoretical selection of items and combining them in accordance with the theoretical concept. Methodologist would label such an operationalization as an ‘index’ rather than a ‘scale’ since the latter implies checking the internal consistency. The quality of an index is usually tested by evaluating its external validity, a principle that is advocated by Welzel and Inglehart (2016) as well. Obviously, there is nothing wrong with theoretical selection of items to measure concepts. After all, this is what all researchers do, even researchers following the ‘dimensional logic’. We subscribe to the idea that creating an index with items measuring *different* aspects of an overarching concept does not require that the constituting aspects intercorrelate consistent across cultures. Education, income and occupation, for instance, can be combined in an index of socio-economic status regardless whether and how these three aspects correlate. But when multiple items are selected to measure these *constituting* parts of the overarching concept, this is another story. In this study we will show that it is important to research what is in the mind of people when answering survey questions. After all the theoretical relevance of selecting items to measure a concept is in the mind of the social scientist. It remains to be seen whether it is also in the mind of the respondent. Imagine the situation in which a researcher would have chosen the items “a job is alright but what most women really want is a home and children” and “having a job is the best way for a woman to be an independent person” to create an index of job-related emancipative values. From a theoretical point of view the first item is a contra-indicative measure and the second an indicative measure. One can easily create an index consistent with this theoretical conceptualization and calculate country means. How the two items relate to one another across countries is then ignored. In this study we take particular interest in researching such variation in associations since it expresses cultural diversity in its own right. Cultures may differ in how they perceive emancipative values. To be sure that differences in associations between items reflect differences in perception of emancipative values we need to recognize that cultural differences in how respondents answer to survey questions may be partially explainable by response style behavior.

### Modeling acquiescence

Acquiescence is a response style that occurs when respondents answer agreeingly to Likert-type items regardless of item content (Billiet and McClendon 2000; Paulhus 1991). It can be caused by, for example, an interaction between an acquiescent personality and a questionnaire that is taxing to the respondent (Baumgartner and Steenkamp 2001), and it also occurs more often among low educated people and older people (Billiet and McClendon 2000; Greenleaf 1992; Meisenberg and Williams 2008; Weijters et al. 2010). Acquiescence can also be regarded as a cultural characteristic, as respondents from different countries and cultures display different levels of acquiescence (Baumgartner and Steenkamp 2001; Diamantopoulos et al. 2006; Greenleaf 1992). For example, van Herk et al. (2004), find that the Mediterranean countries Spain, Italy and Greece have higher levels of acquiescence than the Western European countries Germany, France and England.

The differences in acquiescence can affect the scores and the associations between items differently in each country. Disregarding these effects confounds the identification of cultural differences of content factors with cultural variations in response style behavior. Cheung and Rensvold (2000) and Leung (1989) demonstrate that acquiescence inflates factor scores and intercepts, and consequently leads to scale displacement. For example, it may be that country A and country B have the same true, unbiased score on a certain cultural dimension. They are, for example, equally traditional concerning their family values. However, the respondents in country A score higher on acquiescence, i.e., they answer systematically more agreeing than respondents in country B. Consequently, country A will get a structurally higher, more inflated score on the construct of interest than country B if response style behavior is ignored. In the case of family values, it would seem that country A would have values that are more modern than country B, but this is a difference caused by cultural differences in the acquiescence response style.

Besides the effects of acquiescence on the scores, the latent structure of the data is also affected. Correlations and covariances between items may be inflated, deflated or partly artificial due to spurious relations between variables caused by acquiescent response style behavior (Couch and Keniston 1960; Ray 1983). This, in turn, influences the conclusions about the relationships between these items and scales when, for example, performing factor analysis (Baumgartner and Steenkamp 2001). The standard approach ignores the acquiescence response style in comparative research, making it unclear whether the found differences are cultural differences in content or cultural differences in the use of acquiescence response style. Accounting for acquiescence in the measurement model is therefore essential.

**Fig. 1:**
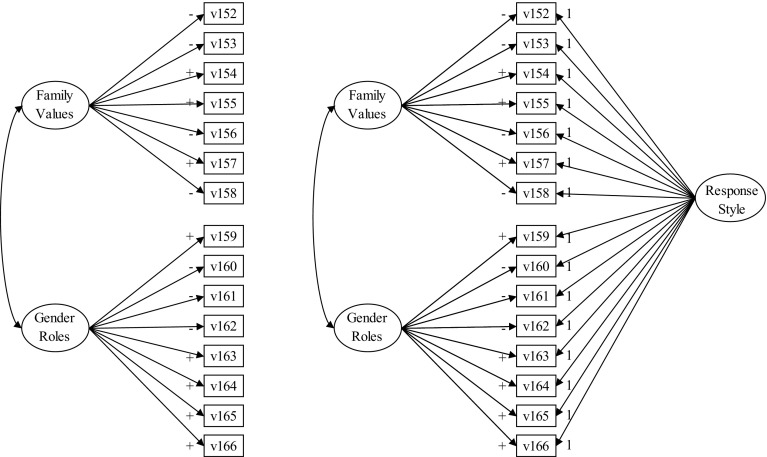
Conceptual CFA models of family values and gender roles, without and with an added response style

A number of different approaches for dealing with acquiescence in Likert-type scales has been developed (Van Vaerenbergh and Thomas 2012). Most of them have in common that balanced sets of items, with both positively and negatively formulated questions, are required in order to uncover acquiescence. With only positively (or only negatively) formulated items, one cannot make a distinction between respondents who acquiesce and respondents who do not acquiesce but agree with the measured constructs (Billiet and McClendon 2000; Paulhus 1991; Ray 1983). However, balancing scales does not necessarily eliminate all variance due to acquiescence, so even with balanced scales acquiescence needs to be controlled. The best method to deal with acquiescence when item sets include both positively and negatively formulated questions has been introduced by Billiet & McClendon (2000; see also Welkenhuysen-Gybels et al. 2003). It involves a confirmatory factor analysis model of two distinct content factors with factor loadings on their sets of items and a latent acquiescence factor that loads on all of the items of the two constructs (Fig. 1). There are two main assumptions regarding acquiescence in this model. Firstly, acquiescence is a common factor behind one or two independent sets of (partially) balanced agree–disagree items. Secondly, acquiescence should have nonzero variance, though its variance should be smaller than the variance of the content factor. Billiet and McClendon (2000) test different models with Belgian data, and Cambre et al. (2002) test the models with European data, and in their studies they find that the model with two latent content factors and one latent acquiescence factor has the best fit in comparison to the model without the acquiescent factor.

In this research we use this approach by comparing a two content factor model distinguishing ‘family values’ and ‘gender roles’, with a model that adds an acquiescence response style (Fig. 1). Both sets of items include positively and negatively worded items (see Table 1). Although it is not a requirement of the method, we have restricted the loadings of the acquiescent response style to be equal across all items for theoretical reasons. If acquiescence constitutes a response ‘style’ rather than a ‘nuisance’, then it should define a constant factor influencing the answering tendencies of respondents to items equally, irrespective of their content. Furthermore, by imposing equality constraints of the acquiescence factor on all items we avoid confounding its measurement with any hidden content in the data, while with unequal loadings this response style factor might capture some obscure ‘content’. Two additional restrictions apply to our models. Firstly, we define the model in such a way that the style factor does not correlate with the latent constructs family values and gender roles, because acquiescence should be independent of one’s opinion on family life matters. The two content factors are allowed to correlate. Secondly, the variances of the latent variables are restricted to be 1, as this automatically standardizes the factor loadings.

**Table 1:** Items for family values and gender roles

Family values (5-point Likert-scale)
v152: a man has to have children in order to be fulfilled (−)
v153: a marriage or a long-term stable relationship is necessary to be happy (−)
v154: homosexual couples should be able to adopt children (+)
v155: it is alright for two people to live together without getting married (+)
v156: it is a duty towards society to have children (−)
v157: people should decide for themselves whether to have children or not (+)
v158: when a parent is seriously ill or fragile, it is mainly the adult child’s duty to take care of him/her (−)
Gender roles (4-point Likert-scale)
v159: a working mother can establish just as warm and secure a relationship with her children as a mother who does not work (+)
v160: a pre-school child is likely to suffer if his or her mother works (−)
v161: a job is alright but what most women really want is a home and children (−)
v162: being a housewife is just as fulfilling as working for pay (−)
v163: having a job is the best way for a woman to be an independent person (+)
v164: both the husband and wife should contribute to household income (+)
v165: in general, fathers are as well suited to look after their children as mothers (+)
v166: men should take as much responsibility as women for the home and children (+)

‘+’ indicates positive worded items, ‘−’ indicates negative worded items

### Data

In this paper we use the data of the European Values Study (EVS) 2008. This is the fourth, most recent wave of the EVS, in which 47 European countries participated,^1^ with a total sample size of 67,786 respondents. Quality control of the data is established by harmonizing data collection and translation across countries. The data was collected by a representative multi-stage or stratified random sample and all the interviews where done face-to-face, with the exception of Finland (internet panel) and Sweden (postal survey). Comparability of constructs can be compromised by the translation of the data (Harkness 2003). For that reason, careful translation of the English source questionnaire in each of the country’s national language was aimed for. Most of the countries used WebTrans, which is a questionnaire database and translation system designed by Gallup (GESIS 2010).

We selected two balanced sets of items that measure family values (7 items) and gender roles (8 items) (see Table 1 for details). The original answering scale of the family values scale ranges from 1 (agree strongly) to 5 (disagree strongly), and the answering scale of the gender roles ranges from 1 (agree strongly) to 4 (disagree strongly). The ‘+’ behind the items are the positively formulated questions, indicating more modern views on the subjects of family values and gender roles, while the ‘−’ items are the negatively formulated questions, indicating the more traditional views. To facilitate interpretation of results we reversed the coding of the items so that a high score corresponds with agreement with the statement.

Respondents with more than two missing values per construct set were omitted from the analysis. This involved 3184 (4.7 %) respondents. Single imputation was used in other cases to avoid further loss of data due to the listwise deletion of respondents. The imputed values were estimated by means of a multinomial logistic regression model using the 15 items from family values and gender roles, and the covariates country, gender, age, and educational level. The final sample size in the analysis consists of 64,602 respondents.

## Method

To identify internally homogeneous groups of countries, we clustered the 47 countries based on their variance-covariance matrices of the 15 items on gender roles and family values. To this end we created a dataset with 47 rows—the 47 countries—and with 120 columns, which consist of the 120 values in each variance-covariance matrix. Each of the variables in this created dataset represents either the variance of an item or the covariance between two items referring to family values and gender roles. We adopted a hierarchical Ward clustering procedure with squared Euclidian distances. The Ward cluster method matches cases in a cluster in such a way that the within-cluster variance is minimized, and it thus clusters countries that are closest together first. The purpose of this first step in the analyses is to define ‘clusters of cultures’ in their similarity regarding the covariance structure in the item sets.

In the next step of the analysis, we conducted two confirmatory factor analyses per found cluster, one excluding and one including acquiescence. In this way we can uncover differences in the measurement models of family values and gender roles between the clusters. By adding acquiescence to the model, we research its impact on the measurement model and again compare the found clusters. It is important to note that no formal measurement invariance testing is conducted neither between nor within clusters. Running separate analyses implies non-invariant measurement models and that is exactly what we aim at since the pre-grouping of countries by means of cluster analysis is expected to maximize the differences in measurement models between clusters. The items in the analysis are ordinal and skewed; we therefore use WLS estimation based on polychoric correlations, as this method gives robust estimates with this type of data (Flora and Curran 2004). Model comparisons are made using the suitable fit indices. We use RMSEA and comparative fit index (CFI), as suggested by Hutchinson and Olmos (1998) since Chi square measures tend to have poor fit with non-normal data and large sample sizes, as is the case in this study. RMSEA values below 0.05 indicate good fit, and below 0.08 there is acceptable fit. The higher the CFI value the better the model fit and preferably its value should exceed the 0.95 level.

Our primary interest is in comparing the results from models with different clusters of cultures, with and without acquiescence. To that purpose we will first research which clusters of countries emerge from comparing their covariances between the selected items. At each level of the cluster classification we define our measurement models with and without acquiescence. Finally country means in each of these models are estimated and compared to evaluate the impact of recognizing ‘clusters of cultures’ in relative position of countries on family values and gender roles.

## Results

### Cluster analysis

The first step in the analysis concerns the a-priori clustering of the 47 European countries in terms of their (dis)similarities in the covariance matrix of gender roles and family value items. The Ward cluster method initiates the analysis with the 47 countries in 47 clusters, which is the cluster solution where all the clusters are internally perfectly homogeneous, because they only contain one country each. One by one, the clusters or countries with the smallest distance are merged, until there is only one cluster left with all 47 countries, and it is maximally heterogeneous.

**Fig. 2:**
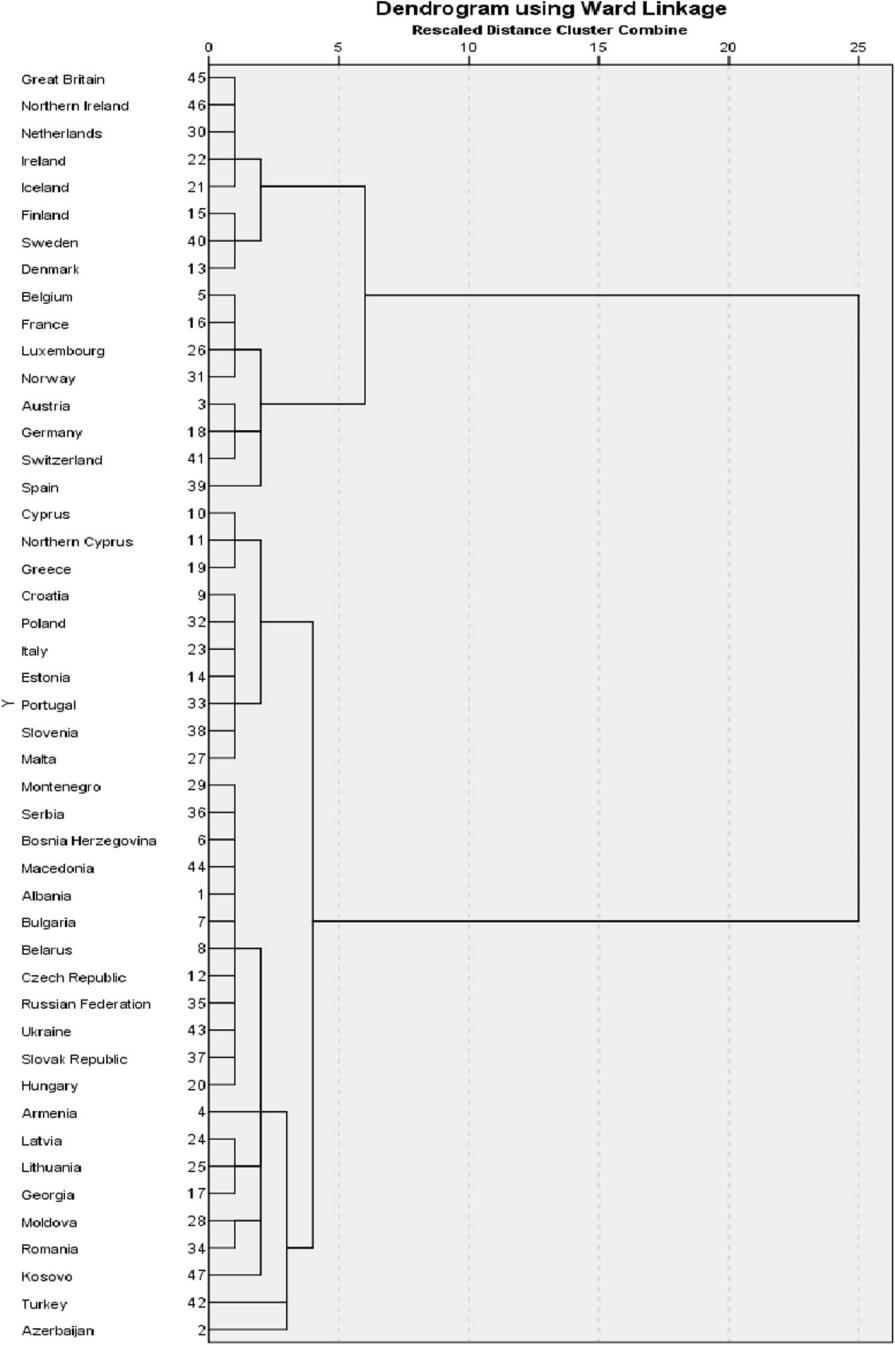
Dendrogram of the ward linkage, squared euclidian cluster analysis

To help decide on the number of clusters to extract, we use the dendrogram of the cluster analysis (Fig. 2). The two cluster solution in the dendrogram identifies a roughly principal distinction between mostly Western versus mostly Eastern European countries. The three-cluster solution further subdivides the Western cluster into a North Western cluster, that includes the British Isles, The Netherlands, and the Nordic countries except for Norway, and a Mid-Western cluster that includes Norway and countries from the mid-west of Europe like France, Germany, Switzerland and Spain. Next, the four cluster solution subdivides the Eastern cluster into a mixed Mediterranean-East cluster, and a Former-Communist cluster. The mixed Mediterranean-East cluster consists of Portugal and the Mediterranean countries Italy, Greece, Malta and (Northern) Cyprus, and also the former-Communist countries Estonia, Poland, Croatia and Slovenia. The Former-Communist cluster consists of countries that used to have a communist regime, like Czech Republic, Latvia, former Yugoslavian states and Russia. Turkey is the exception in this cluster, as it never had a communist regime.

Although subsequent cluster solutions further subdivide the initial cluster compositions, and hence increasingly define more homogeneous clusters of countries, we decided to not further increase the number of clusters for two reasons. First, additional clusters merely implied further fragmentation of countries into either outliers (a single country defining a cluster) or a small number of countries. Second, the main source of incomparability among groups of countries would be observed in subdividing the pool of countries into two and four clusters, whereas further subdividing these clusters would reflect secondary sources of differences in measurement model. The logic of this reasoning will be revealed when interpreting the results. We will show that the biggest change in country location on family values and gender role is when comparing the pooled (1-cluster) measurements with the 2-cluster model. Differences are smaller when comparing the 2-cluster with the 4-cluster model.

### Confirmatory factor analyses

The focus of these analyses is to compare measurement models across clusters and to investigate the impact of acquiescence. 14 different models are tested and the fit indices compared, as is presented in Table 2. The first two models in Table 2 are based on the complete sample of all 47 European countries. The next four models are based on the two-cluster solution, with the Western cluster (Models A) and the Eastern cluster (Models B). Both of these clusters are modeled without and with acquiescence (respectively Models A−/B− and Models A+/B+). The next eight models are based on the four-cluster solution. The Western cluster subdivides in a North Western cluster (A1) and a Mid-Western cluster (A2), and the Eastern cluster subdivides in a Mediterranean-East cluster (B1) and a Former-Communist cluster (B2). Again, the ‘−’ and ‘+’ behind the model number indicates the exclusion or inclusion of acquiescence in the model. All our models display a RMSEA below 0.05, indicating good fit for all the models that are tested. However, the CFI never reaches the benchmark of 0.95. Part of this is due to the fact that not all items act in an optimal and consistent way across models. Factor loadings—presented further in Table 3—show that particular items have relatively low loadings depending on the model estimated. The consequence of that is that CFI indices deteriorate. The usual solution is to delete such items from the model but in the current study this is not an option since identifying items that caused poorer CFI values varies from model to model. This already shows the diversity in meaning given to the items depending on which countries are included in the measurement model. The fit measures in this paper do however indicate the improvement of the fit when adding the response style acquiescence to the model and when moving from one, to two, to four European clusters.

**Table 2:** Model fit of the estimated models

Cluster (number of countries)	Model	Baseline model		Model fit		RMSEA			CFI
		χ^2^	*df*	χ^2^	*df*	Estimate	90 % CI		
1 European cluster, single group CFA									
All countries (47)	No RS	4332.08	105	2934.14	89	0.022	0.022	0.023	0.327
	With RS	4332.08	105	1791.99	88	0.017	0.016	0.018	0.604
2 European clusters, single group CPA									
A− Western cluster (16)	No RS	4847.537	105	1225.516	89	0.025	0.024	0.026	0.760
A+	With RS	4847.537	105	901.935	88	0.021	0.020	0.023	0.828
B− Eastern cluster (31)	No RS	3795.647	105	1854.371	89	0.021	0.020	0.022	0.522
B+	With RS	3795.647	105	1341.314	88	0.018	0.017	0.019	0.660
4 European clusters, single group CPA									
Al− North Western cluster (8)	No RS	2684.709	105	679.737	89	0.028	0.026	0.030	0.771
A1+	With RS	2684.709	105	473.456	88	0.022	0.021	0.024	0.851
A2− Mid Western cluster (8)	No RS	3442.333	105	924.401	89	0.028	0.027	0.030	0.750
A2+	With RS	3442.333	105	617.000	88	0.023	0.021	0.024	0.841
B1− East-Mediterranean cluster (10)	No RS	3107.834	105	1780.742	89	0.038	0.037	0.040	0.437
B1+	With RS	3107.834	105	948.365	88	0.027	0.026	0.029	0.713
B2− Former-Communist cluster (21)	No RS	2764.342	105	1101.156	89	0.019	0.018	0.020	0.619
B2+	With RS	2764.342	105	840.174	88	0.017	0.016	0.018	0.717

A. Western cluster	A1: North Western cluster	A2: Mid-Western cluster
	Denmark	Austria
	Finland	Belgium
	Great Britain	France
	Iceland	Germany
	Ireland	Luxembourg
	Netherlands	Norway
	Northern Ireland	Spain
	Sweden	Switzerland

B. Eastern cluster	B1: Mediterranean-East cluster	B2: Former-Communist cluster	
	Croatia	Albania^a^	Armenia^b^
	Cyprus	Belarus^a^	Georgia^b^
	Estonia	Bosnia Herzegovina^a^	Latvia^b^
	Greece	Bulgaria^a^	Lithuania^b^
	Italy	Czech Republic^a^	Moldova^b^
	Malta	Hungary^a^	Romania^b^
	Northern Cyprus	Macedonia^a^	Kosovo^b^
	Poland	Montenegro^a^	
	Portugal	Russian Federation^a^	Azerbaijan
	Slovenia	Serbia^a^	Turkey
		Slovak Republic^a^	
		Ukraine^a^	

The parameters from the response style are restricted to be equal across items

P-values of χ^2^ of all models is 0.000, P close of all models is 1.000

No RS/with RS: models without added response style in the model, and models with added response style in the model

^a^Subsample B2_12

^a,b^Subsample B2_19

**Table 3:** Parameter estimates of the two and four-cluster model, with and without the acquiescence response style

Variables	All countries		A: Western cluster		A1: North Western cluster		A2: Mid Western cluster	
	T−: no RS	T+: with RS	A−: no RS	A+: with RS	A1−: no RS	A1+: with RS	A2−: no RS	A2+: with RS
	Estimate (SE)	Estimate (SE)	Estimate (SE)	Estimate (SE)	Estimate (SE)	Estimate (SE)	Estimate (SE)	Estimate (SE)
FV BY								
V152	0.662 (0.031)	0.664 (0.031)	−0.476 (0.054)	−0.512 (0.053)	−0.506 (0.066)	−0.493 (0.063)	−0.663 (0.029)	−0.650 (0.029)
V153	0.540 (0.019)	0.563 (0.018)	−0.502 (0.019)	−0.496 (0.018)	−0.415 (0.024)	−0.465 (0.024)	−0.572 (0.014)	−0.545 (0.013)
V154	−0.474 (0.029)	−0.441 (0.030)	0.506 (0.015)	0.559 (0.016)	0.521 (0.020)	0.527 (0.020)	0.477 (0.020)	0.518 (0.020)
V155	−0.671 (0.022)	−0.691 (0.023)	0.740 (0.015)	0.703 (0.016)	0.821 (0.028)	0.775 (0.029)	0.686 (0.017)	0.686 (0.017)
V156	0.554 (0.029)	0.594 (0.029)	−0.572 (0.021)	−0.600 (0.020)	−0.560 (0.028)	−0.598 (0.027)	−0.586 (0.022)	−0.622 (0.022)
V157	−0.413 (0.026)	−0.414 (0.026)	0.612 (0.024)	0.574 (0.025)	0.663 (0.042)	0.625 (0.041)	0.613 (0.024)	0.591 (0.024)
V158	0.249 (0.044)	0.328 (0.044)	−0.263 (0.022)	−0.304 (0.019)	−0.280 (0.018)	−0.367 (0.020)	−0.216 (0.029)	−0.269 (0.029)
GR BY								
V159	0.447 (0.025)	0.400 (0.027)	0.586 (0.016)	0.571 (0.016)	0.693 (0.018)	0.667 (0.017)	0.571 (0.020)	0.581 (0.019)
V160	−0.307 (0.028)	−0.561 (0.024)	−0.644 (0.026)	−0.718 (0.025)	−0.665 (0.015)	−0.780 (0.015)	−0.687 (0.030)	−0.748 (0.032)
V161	−0.205 (0.021)	−0.503 (0.018)	−0.488 (0.021)	−0.570 (0.019)	−0.496 (0.030)	−0.588 (0.030)	−0.513 (0.028)	−0.553 (0.027)
V162	−0.140 (0.019)	−0.350 (0.018)	−0.280 (0.016)	−0.319 (0.016)	−0.278 (0.022)	−0.298 (0.021)	−0.326 (0.017)	−0.334 (0.017)
V163	0.493 (0.015)	0.367 (0.019)	0.404 (0.018)	0.361 (0.019)	0.336 (0.027)	0.309 (0.027)	0.389 (0.029)	0.377 (0.031)
V164	0.615 (0.018)	0.436 (0.021)	0.490 (0.027)	0.434 (0.029)	0.373 (0.025)	0.347 (0.023)	0.515 (0.037)	0.474 (0.041)
V165	0.629 (0.010)	0.489 (0.013)	0.630 (0.010)	0.579 (0.012)	0.609 (0.013)	0.573 (0.012)	0.628 (0.014)	0.580 (0.016)
V166	0.721 (0.012)	0.569 (0.015)	0.717 (0.014)	0.656 (0.015)	0.696 (0.021)	0.641 (0.021)	0.700 (0.020)	0.621 (0.019)
RS BY								
v152–v166		−0.354 (0.008)		0.264 (0.010)		−0.253 (0.004)		−0.254 (0.009)
GR with FV	−0.332 (0.039)	−0.467 (0.036)	0.702 (0.016)	0.661 (0.018)	0.639 (0.022)	0.623 (0.020)	0.670 (0.025)	0.640 (0.026)

Variables	B: Eastern cluster		B1: East-Mediterranean cluster		B2: Former-Communist cluster	
	B−: no RS	B+: with RS	B1−: no RS	B1+: with RS	B2−: no RS	B2+: with RS
	Estimate (SE)	Estimate (SE)	Estimate (SE)	Estimate (SE)	Estimate (SE)	Estimate (SE)
FV BY						
V152	0.549 (0.019)	0.581 (0.017)	−0.404 (0.033)	0.571 (0.028)	0.572 (0.020)	0.515 (0.022)
V153	0.609 (0.025)	0.580 (0.022)	−0.422 (0.039)	0.514 (0.036)	0.626 (0.027)	0.531 (0.027)
V154	−0.072 (0.024)	−0.455 (0.025)	0.329 (0.037)	−0.324 (0.038)	−0.059 (0.029)	−0.498 (0.035)
V155	0.029 (0.042)	−0.374 (0.037)	0.753 (0.026)	−0.596 (0.021)	0.055 (0.052)	−0.287 (0.055)
V156	0.414 (0.021)	0.314 (0.020)	−0.268 (0.026)	0.504 (0.034)	0.435 (0.021)	0.263 (0.019)
V157	0.442 (0.029)	0.125 (0.032)	0.517 (0.027)	−0.436 (0.028)	0.488 (0.032)	0.275 (0.039)
V158	0.585 (0.021)	0.408 (0.035)	−0.052 (0.038)	0.212 (0.038)	0.561 (0.019)	0.405 (0.028)
GR BY						
V159	0.342 (0.025)	0.294 (0.031)	0.475 (0.018)	0.421 (0.020)	0.335 (0.028)	0.257 (0.041)
V160	0.120 (0.028)	−0.271 (0.024)	−0.495 (0.020)	−0.543 (0.022)	0.185 (0.032)	−0.227 (0.032)
V161	0.261 (0.016)	−0.100 (0.015)	−0.376 (0.014)	−0.453 (0.015)	0.315 (0.018)	−0.038 (0.019)
V162	0.170 (0.022)	−0.194 (0.020)	−0.167 (0.029)	−0.333 (0.031)	0.238 (0.026)	−0.166 (0.024)
V163	0.517 (0.015)	0.479 (0.012)	0.516 (0.025)	0.426 (0.032)	0.509 (0.020)	0.478 (0.015)
V164	0.706 (0.010)	0.637 (0.010)	0.571 (0.023)	0.447 (0.029)	0.704 (0.012)	0.681 (0.010)
V165	0.575 (0.020)	0.449 (0.016)	0.523 (0.010)	0.434 (0.011)	0.569 (0.024)	0.434 (0.022)
V166	0.703 (0.017)	0.610 (0.017)	0.666 (0.017)	0.561 (0.022)	0.697 (0.021)	0.610 (0.022)
RS BY						
v152–v166		0.341 (0.009)		0.328 (0.004)		0.350 (0.010)
GR with FV	0.548 (0.020)	0.111 (0.026)	0.413 (0.021)	−0.428 (0.024)	0.608 (0.020)	0.237 (0.030)

The response style is restricted to load equal on all items, the associations between content factors (FV and GR) and response style factor is fixed to 0

*FV* family values, *GR* gender roles, *RS* response style

When the model with all the countries in one cluster is estimated, the CFI with a value of 0.327 is very low, and although the CFI increases to 0.604 when adding the response style, it is obvious that the imposed model does not fit all the European countries pooled together in one cluster. In the next step the fit of both the Western (A) and Eastern (B) clusters is better than in the model with all the countries in one cluster, and the fit improves even more when acquiescence is included into the model, with the Western cluster having a CFI of 0.828 and the Eastern cluster a lower CFI of 0.660. Next, these clusters are split into four distinct clusters, containing an even more homogeneous pool of countries. This further improves model fit, and besides that, the four clusters benefit from the added acquiescence.

The best fitting model is model A1+, the North Western Cluster with acquiescence, while the less well fitting models are found with the Mediterranean-East and Former-Communist clusters. An explanation might be that the items sets were formulated in a Western European context since the items were included in the early waves of the EVS project, and they might not translate well to other cultural (e.g. Eastern European) settings.

In short, what the results indicate is that clustering the countries into clusters that are more internally homogeneous and adding acquiescence to the model improves model fit. In the next section we are going to inspect the estimated factor loadings of these models more closely, and compare the clusters and the models with and without acquiescence.

Table 3 displays the factor loadings of the pooled (T), the 2-clusters (A and B) and 4-clusters analyses (A1, A2, B1, and B2). In all cases a distinction is made between a model without (e.g. A−) and with (e.g. A+) acquiescence. In interpreting factor loadings it is important to recall that reversal of the sign of the loadings from one analyses to the other might occur. This merely reflects reverse scaling of the latent factors and has no further substantive meaning.

The results from the pooled analyses demonstrate what has been indicated in the literature, namely that factor loadings of positively and negatively worded items become more similar in weight when adjusting for acquiescence. Even the association between family values and gender roles increases. More striking is the comparison with the results when analyses are done separately for the two major clusters A and B. The Western (A) cluster’s results are fairly close to the results from the pooled analyses. The Eastern (B) cluster differs profoundly even in such a way that in the model without acquiescence the loadings of all items except one are in the same direction rather than showing opposite signs consistent with the theoretical labeling presented in Table 1. Adding acquiescence reduces the anomaly and partly restores the expected factor loading structure, except for item V157 that remains inconsistent. A second striking finding is that factor loadings of items differ in magnitude between both clusters even when acquiescence is accounted for. The negatively worded Family Values items (V154, V155 and V157) and the positively worded Gender Role items (V160, V161 and V162) show substantive lower loadings in the Eastern cluster. Finally it is worth mentioning that item V158 on the topic of ‘a child’s duty to take care of parents’ has poor loadings in the pooled and Western (A) cluster model. In the Eastern (B) cluster this changes and the item contributes more clearly in defining family values. That these general clusters differ in their perception of family values and gender role is also expressed in the correlation between these two latent factors. In the Western (A) cluster they correlate as expected: being egalitarian in family values correlates with being egalitarian in gender role values. This relationship is not observed in the Eastern (B) cluster: the correlation is weak and not in the expected direction. To clarify this unexpected result we need to have a closer look at the results from the 4-cluster analyses.

The aforementioned findings imply that the presumed balancing of items and their contribution in identifying family values is cultural (pre)defined. Certain items in the set have very specific meanings across both general clusters. It is equally clear from these findings that splitting up the pooled data into two clusters of countries was valuable.

Further subdividing both general clusters again show diversity in results. Comparing factor loading structures of the two sub-clusters (A1 and A2) within the Western (A) cluster shows only minor differences in magnitude of loadings. The subdivision of the Eastern (B) cluster proved to be valuable since the smaller cluster of East-Mediterranean countries (B1) reveal factor loadings that are much more consistent with the theoretically expected signs of loadings. Adding acquiescence even further increases consistency with expectations. The correlation between the two latent factors is also as expected but less strong than in the two Westers clusters. This cluster B1 only differs in magnitude of loadings compared to the Western clusters. The picture of factors presented in the B cluster model was dominated by the countries classified in the Former-Communist (B2) cluster. Interpretations of model B should thus be limited to countries included in model B2.

The results from cluster B2 raise questions since they deviate most from theoretical expectations. Even adding acquiescence only partly resolves the issue. One line of reasoning that we tested was: ‘what happens if we further subdivide cluster B2 into multiple clusters?’ The dendrogram presented in Fig. 2 then reveals that the next step in clustering would first result in isolating two countries that define a cluster of their own, i.e. Turkey and Azerbaijan. Further subdivision results in separating Kosovo from two clusters with a few countries and one larger cluster of 12 countries. We have rerun model B2 analyses with limiting the number of countries included, first excluding Turkey and Azerbaijan (selected countries B12_19 in Table 2), then restricting the analyses to include the selection of 12 countries (selected countries B2_12 in Table 2) with the highest level of homogeneity in their covariance structure. Comparing factor loadings of models without acquiescence revealed no changes in loadings. Changes were only observed in models with acquiescence. In the model excluding Turkey and Azerbaijan the single most important change was with item V157. Although small, the loading was now consistent with theoretical expectation which was not the case in model B2. Further limiting the analyses to the aforementioned selection of 12 countries confirmed the change in loading of V157 and the magnitude of its effect increased substantially to the same level as the two other positively worded family values items (V154 and V155). On top of that the correlation between the two latent class factors became consistent with theoretical expectations. In none of the analyses including the selection of countries from cluster B we observed the same size in association between family values and gender roles as we found in cluster A.

Taken together these analyses clearly demonstrate that the ‘clusters of cultures’ approach developed in this research was capable of detecting major cleavages in measurement models of grouped countries. The approach involved classifying countries according to their similarity in covariances between family values and gender role items. The first clustering into two general clusters revealed the most substantive differences in measurement. Further subdivision of these two general clusters proved to be relevant for only one of them, namely the Eastern (B) cluster. Additionally limiting the selection of countries within one of these sub-clusters (B2) resulted in fine-tuning results: the overall picture is less affected but it accounted for item-specific inconsistencies.

### What difference does it make? Impact of differential ways of measuring family values and gender roles on country differences

As indicated above the adherents to a ‘combinatory logic’ in creating indices of values orientations do not feel a need to establish measurement invariance (Welzel and Inglehart 2016). We argued that combining items following a theoretical logic is not necessarily wrong but needs to comply with what is in the mind of people. For this purpose we present comparisons of (dis)similarities between the different measures of Family Values and Gender roles in two ways: at the level of individual level correlates and by comparing country means. Our point of departure is the classification of countries into four clusters. Per cluster we have computed factor scores from the pooled, the 2-cluster and the 4-cluster analyses and standardized them within each cluster to make them comparable within each of the four clusters. We also calculated a sum-score index that is consistent with the theoretical classification of the items as presented in Table 1. Such a sum-score corresponds with the ‘combinatory logic’ mentioned before. The main difference between this sum-score and the factor scores from the pooled analysis is that the latter uses a weighted combination of items whereas the first assigns an equal weight to all items. In Table 4 we present correlations between the sum score indices and the factor scores. The sum score is the theoretically defined operationalization—that is what is in the mind of the researcher—and the factor scores are what is inferred from the response patterns of respondents—which is in the mind of the individual.

**Table 4:** Correlations of sum-score indices of family values and gender roles with factor scores

Cluster	Pooled (1-cluster)		2-Clusters separate		4-Clusters separate	
	No RS	With RS	No RS	With RS	No RS	With RS
1. Family values (high = egalitarian)						
A1	0.956	0.949	0.888	0.919	0.886	0.918
A2	0.970	0.962	0.919	0.942	0.941	0.947
B1	0.969	0.961	0.680	0.938	0.866	0.959
B2	0.960	0.951	0.602	0.914	0.584	0.854
2. Gender roles (high = egalitarian)						
A1	0.919	0.974	0.935	0.942	0.934	0.929
A2	0.909	0.974	0.941	0.946	0.948	0.948
B1	0.878	0.967	0.591	0.910	0.944	0.972
B2	0.856	0.967	0.579	0.911	0.527	0.875

As far as both Western clusters (A1 and A2) are concerned the correlations are relatively high—although not perfect—regardless whether the correlations were calculated from the pooled versus 2- and 4-cluster model. This suggests that the grouping of countries had little impact. Accounting for acquiescence generally increases these correlations except if they were already at a high level when the response style was not modelled. This is hardly a surprise since we already indicated that the model with ARS produced factor loadings that are more consistent with the theoretical classification of the negatively and positively worded items per latent factor (see Table 3). The story is different when inspecting correlations within the Eastern clusters B1 and B2. A reader should not be surprised in seeing that these correlations are relatively high when they were computed from the pooled factor analyses. After all, the pooled analyses—especially when ARS was included—resulted in factor loadings that were in agreement with the theoretical classification of items. A key finding from Table 3, as indicated before, is that this factor structure is not observed when running analyses separately for Eastern (models B, B1 and B2) clusters. The importance of controlling for acquiescence within the Eastern clusters is also highlighted: when ARS is taken into account correlations substantially increase. The particularity of the B1 versus B2 cluster is highlighted in de findings from the 4-cluster model. Correlations are high within B1 but substantially lower in B2. This means that the discrepancy between theoretical meaning and what is in the mind of people is highest within this cluster. If we keep in mind that the theoretical logic of the item selection comes from within Western (European) tradition—the EVS-study was first set-up within Western European E.U. countries—this finding indicates the family values and gender role items do not necessarily mean the same thing across cultural systems.

**Fig. 3:**
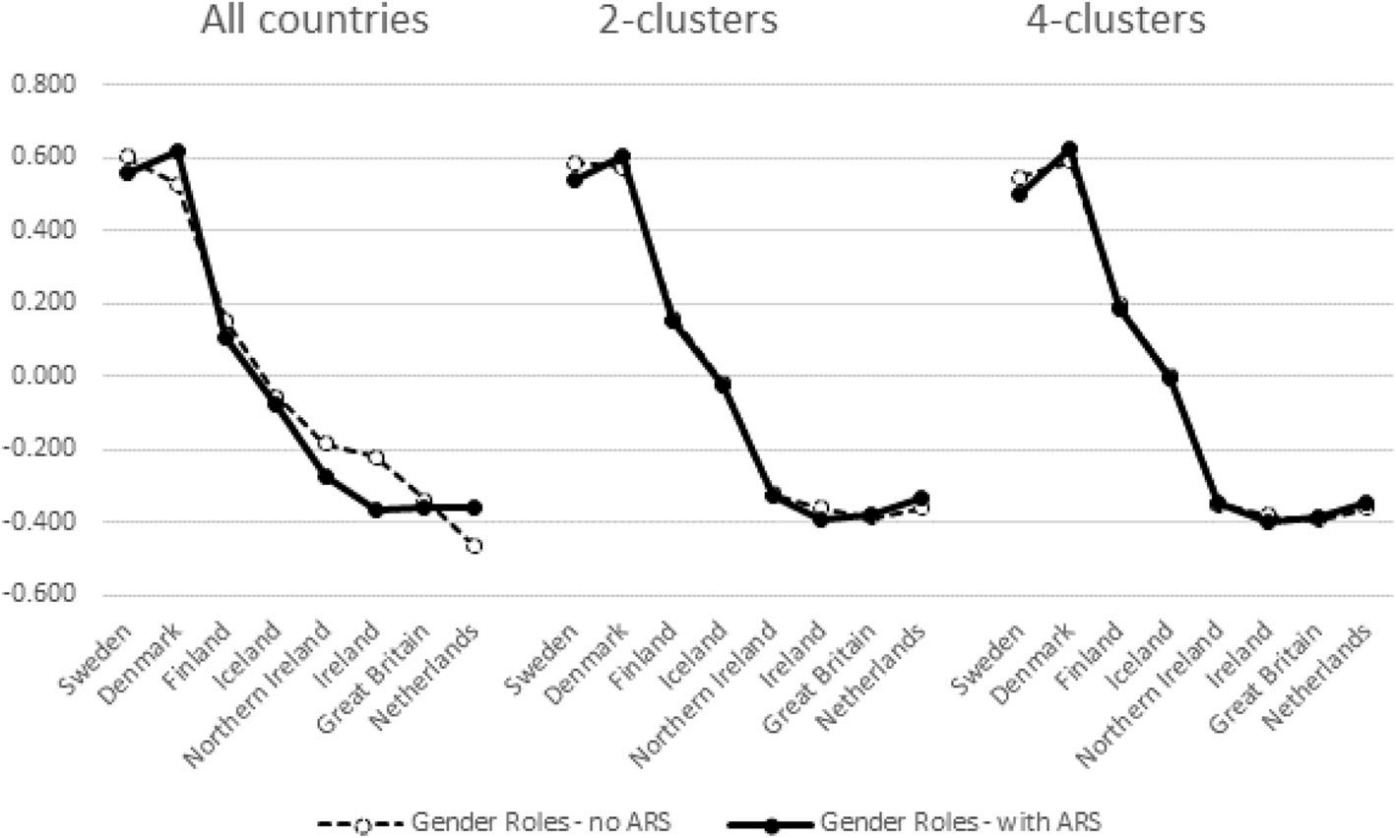
Aggregated mean factor scores of gender roles in the North Western cluster (A1)

**Fig. 4:**
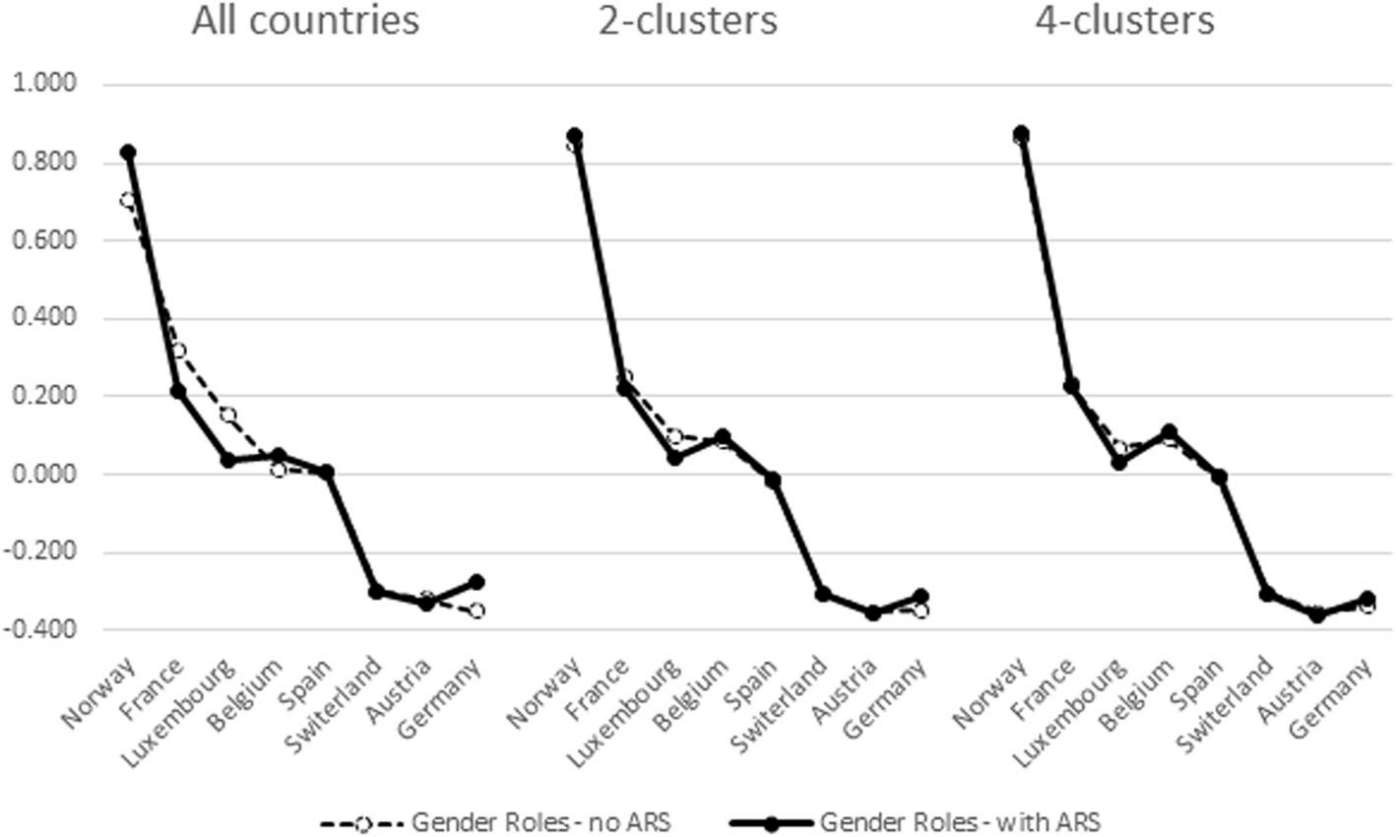
Aggregated mean factor scores of gender roles in the Mid-Western cluster (A2)

**Fig. 5:**
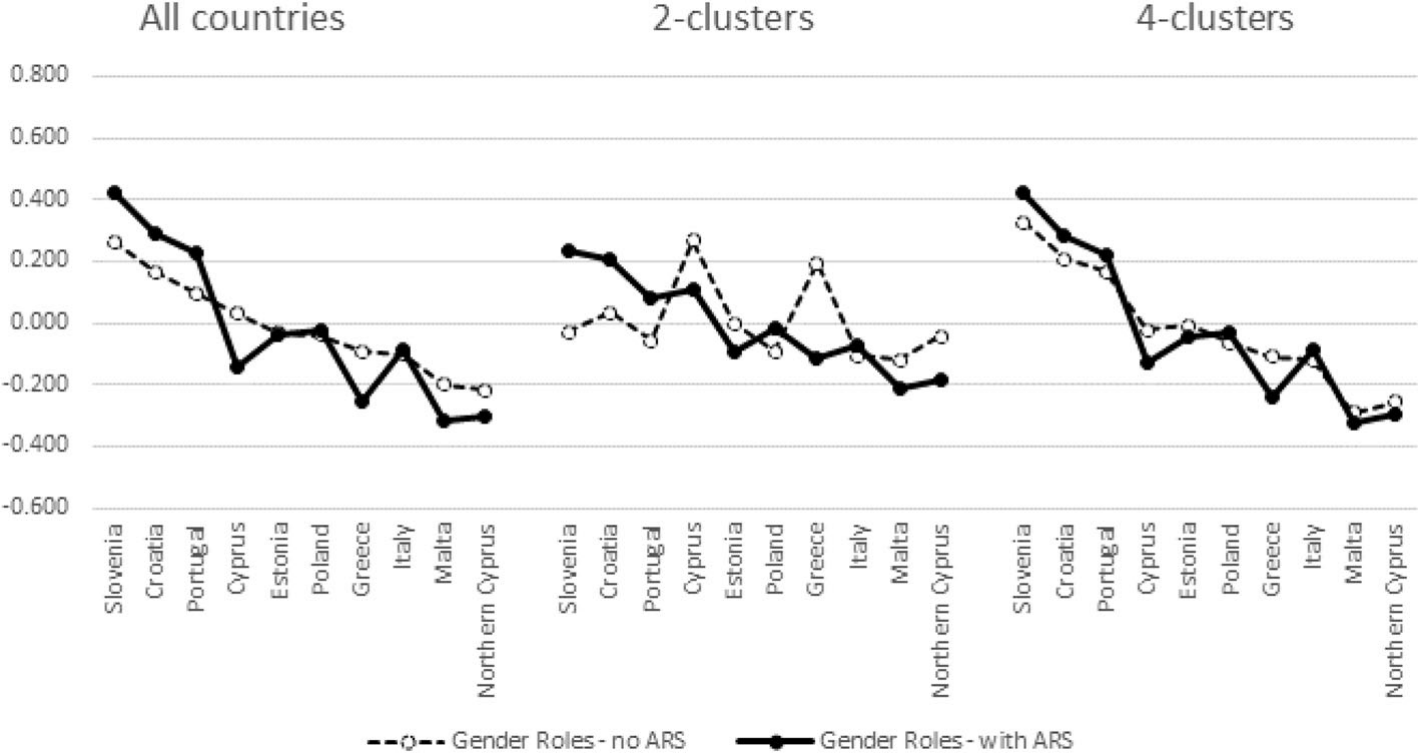
Aggregated mean factor scores of gender roles in the Mediterranean-East cluster (B1)

**Fig. 6:**
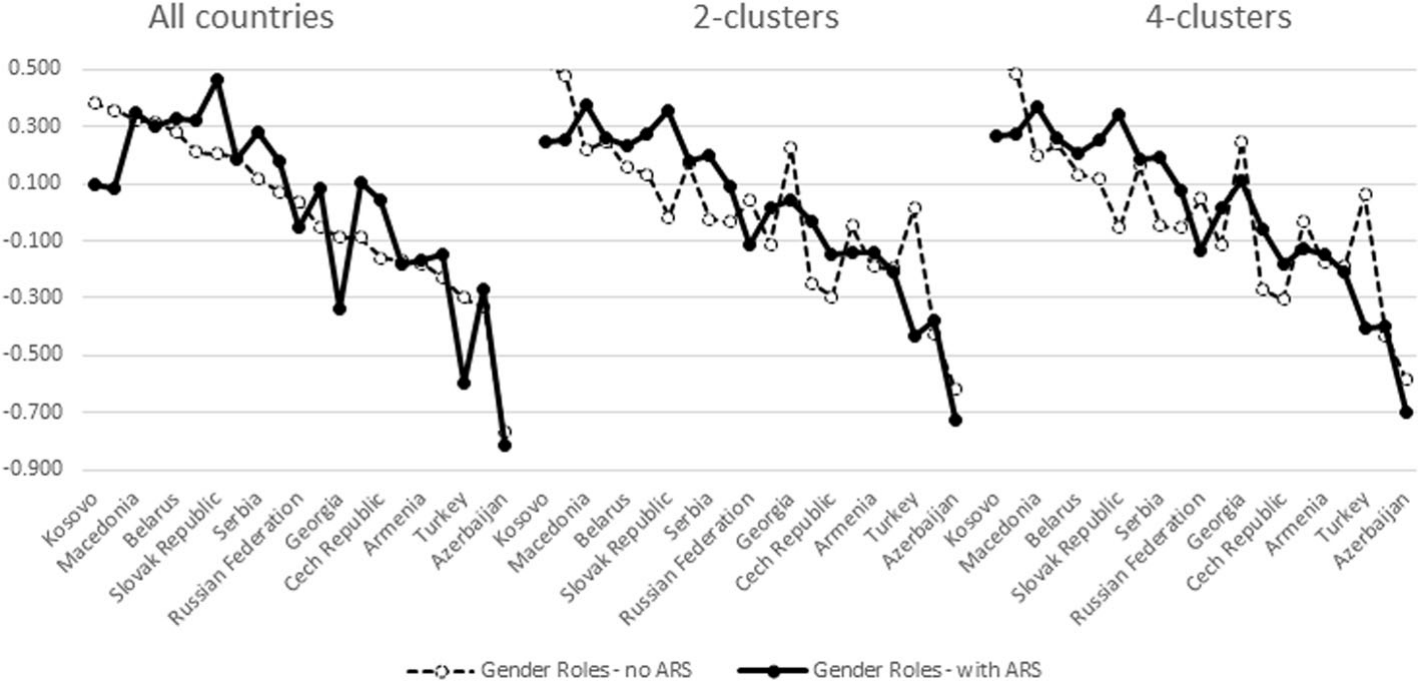
Aggregated mean factor scores of gender roles in the former communist cluster (B2)

These individual level correlations indirectly indicate that country differences in factor mean scores might change depending on which cluster is examined and whether or not ARS is accounted for. Figures 3, 4, 5, and 6 provide the evidence in case of Gender Roles. Similar findings were observed in case of Family Values. We have ordered the countries in descending order of factor scores estimated with the pooled data without ARS. Zigged lines indicate that country order is thus altered compared to this reference. Higher scores indicate higher levels of egalitarianism in gender roles.

We indicated before that the subdivision of the Western (A) cluster into two sub-clusters (A1 and A2) did not reveal major differences in factor weights but that the difference with the pooled results is more important. In Figs. 3 and 4 this interpretation is substantiated. Figures from the 2- versus 4-cluster solution hardly differ. The principal differences with the pooled results are that (a) the 4 Nordic countries are separated more clearly from the other countries in Cluster A1—with Nordic countries being more egalitarian in gender roles; and (b) Belgium (cluster A2) being located higher on gender roles in the cluster specific analyses than in the pooled analyses. These findings apply most when ARS is taken into account since differences in mean factor loadings in the pooled data are clearly affected by it. ARS doesn’t impact on country means in the cluster-specific analyses.

Contrary to the Western clusters ARS clearly impacts on country means of the Eastern clusters. This finding is in line with findings from the measurement models (Table 3) as well as with results from calculating individual-level correlations between factor-scores (Table 4). That separating cluster B1 from B2 was relevant is highlighted by the finding that the results from the 2-cluster analysis (pooling all countries from B1 and B2) produces country differences within cluster B1 that are clearly different from the separate analyses in the 4-cluster model. Country differences within cluster B1 are similar irrespective of whether they are obtained from the pooled or from the 4-cluster model. The particularity of cluster B2 results is illustrated in Fig. 6. In this case the 2-cluster and 4-cluster results are more in line, thus confirming our previous interpretation that the 2-cluster measurement model was dominated by the countries classified in cluster B2. The zigged lines illustrate a strong impact of ARS on country location. What is more important is that country differences within this cluster of former communist countries vary depending whether they are estimated from the pooled data versus the cluster-specific results. This is definitely the case when ARS is not taken into account, but even controlling for ARS leads to differential location of countries on their mean gender roles values. Again this is in line with the finding that at the individual level the correlation of the factor scores with the gender role index is lowest (0.875, see Table 3). The implication of this finding is that the alleged theoretical interpretation of the meaning of items is not unequivocally reflected in the mind of the respondents belonging to this cluster B2.

## Summary and discussion

This research set out to explore cultural variations in the meaning that is assigned to items indicating family values and gender roles. We focus on two different complications that impact on the responses given to two sets of items from the EVS of 2008. One complication is the variation in content meaning given to items as is expressed in the underlying covariation among items. The second complication is defined by cultural variations in how respondents answer survey questions independent of the content that is being measured. Our exploratory journey started with a cluster analysis of the country specific (co)variance matrices which allowed us to classify countries in clusters of cultures that differ in their (co)variances between items. This proved to be a valuable approach since the major difference between cultures was articulated in the comparison of the separate measurement models across two general and four subdivided clusters with results from the pooled (one group) analyses. Most striking was the finding that the assumed balancing of items was not observed in all situations. This balancing was partly restored by adding an acquiescence response style. Measurement models differed across cultures. We advise that applied researchers show some reluctance in interpreting the family values and gender role items as representing the same concept across cultures. Our research showed that this is an assumption that is not tenable in all situations. A second finding with implications for cross-cultural comparative research is that ranking countries on family values or gender roles may be spurious due to response style behavior. This finding is not unique to our research but we discovered that acquiescence only affected country rankings in the Eastern countries and more specifically countries classified within the Former-Communist cluster.

This study introduced the idea that ‘clusters of countries’ can be identified by comparing differences in covariance structures in a set of items intended to measure family values and gender roles. Respondents from countries that share a similar covariance structure tend to think alike in responding to the survey items. That thinking is (or might be) linked to both the content of the items and the response style respondents may use when answering survey questions. Our analyses have demonstrated the usefulness of this approach. Obviously there are limits to the clustering of countries: the more groups defined, the less interesting the within cluster comparisons become since they only pertain to few countries. In this research moving beyond the 4-cluster solution implies finding ‘clusters’ of separate countries and clusters including 2 or 3 countries. What was interesting is that moving beyond the 4-cluster solution only suggested that one particular item’s meaning substantially changed by omitting particular countries from the pool together with one association. Hence, the difference was in details rather than in the overall picture.

There are challenges to future research that emerge from this study. We clearly demonstrated that accounting for acquiescence has the potential to increase comparability in measurement between cultural clusters as well as to impact on country differences in the attitudes or values of interest, in this case: gender roles and family values. In surveying the literature, we found few datasets that include (partially) balanced scales of items; a prerequisite to model acquiescence. A first challenge then is to define such balanced sets of items to measure different attitudes and values. However, in some cases, it might not be possible to define balanced sets of items. In these instances, the method used in our approach that disentangles covariance of items into common content versus response style, is not applicable. A second challenge to future research is therefore to investigate whether a measure of acquiescence can be found that is not based on the response behavior portrayed by these balanced scales, but still accommodates for response biases as well, thus having a positive impact on cross-cultural comparative research. By extension this applies to the idea that other types of response style behavior might have similar impact on the measurement of attitudes or values. Further digging into how to measure response style behavior in order to improve measurement models, thus improving the substantive conclusions resulting from cross-cultural comparisons is definitely subject of our future research.

## Footnotes

1 The 47 European countries with sample size are: Albania (1534), Armenia (1500), Austria (1510), Azerbaijan (1505), Belarus (1500), Belgium (1509), Bosnia and Herzegovina (1512), Bulgaria (1500), Croatia (1525), Cyprus (1000), Czech Republic (1821), Denmark (1507), Estonia (1518), Finland (1134), France (1501), Georgia (1500), Germany (2075), Great Britain (1561), Greece (1500), Hungary (1513), Iceland (808), Ireland (1013), Italy (1519), Kosovo (1601), Latvia (1506), Lithuania (1500), Luxembourg (1610), Republic of Macedonia (1500), Malta (1500), Republic of Moldova (1551), Republic of Montenegro (1516), Netherlands (1554), Northern Cyprus (500), Northern Ireland (500), Norway (1090), Poland (1510), Portugal (1553), Romania (1489), Russian Federation (1504), Serbia (1512), Slovak Republic (1509), Slovenia (1366), Spain (1500), Sweden (1187), Switzerland (1272), Turkey (2384), Ukraine (1507). The total sample size of the study is 67,786.
